# Deep Brain Stimulation for Focal or Segmental Craniocervical Dystonia in Patients Who Have Failed Botulinum Neurotoxin Therapy—A Narrative Review of the Literature

**DOI:** 10.3390/toxins15100606

**Published:** 2023-10-09

**Authors:** Thorsten M. Odorfer, Jens Volkmann

**Affiliations:** Department of Neurology, University Hospital Würzburg, Josef-Schneider-Str. 11, 97080 Würzburg, Germany

**Keywords:** cervical dystonia, Meige syndrome, deep brain stimulation, internal globus pallidus, subthalamic nucleus, botulinum neurotoxin, medication therapy failure, symptom control, safety and tolerability

## Abstract

(1) Background: The first-line treatment for patients with focal or segmental dystonia with a craniocervical distribution is still the intramuscular injection of botulinum neurotoxin (BoNT). However, some patients experience primary or secondary treatment failure from this potential immunogenic therapy. Deep brain stimulation (DBS) may then be used as a backup strategy in this situation. (2) Methods: Here, we reviewed the current study literature to answer a specific question regarding the efficacy and safety of the use of DBS, particularly for cervical dystonia (CD) and Meige syndrome (MS) in patients with documented treatment failure under BoNT. (3) Results: There are only two studies with the highest level of evidence in this area. Despite this clear limitation, in the context of the narrowly defined research question of this paper, it is possible to report 161 patients with CD or MS who were included in studies that were able to show a statistically significant reduction in dystonic symptoms using DBS. Safety and tolerability data appeared adequate. However, much of the information is based on retrospective observations. (4) Conclusions: The evidence base in this area is in need of further scientific investigation. Most importantly, more randomized, controlled and double-blind trials are needed, possibly including a head-to-head comparison of DBS and BoNT.

## 1. Introduction

The clinical symptom dystonia describes an involuntary contraction of the muscles or muscle groups, resulting in abnormal, sometimes even bizarre movements or postures of single or multiple joints. Under the umbrella term dystonia, various disease entities are subsumed, whose common feature is the aforementioned movement disorder. Dystonias are heterogeneous in their clinical manifestations apart from this defining feature, and to some extent, in their underlying pathogenesis and pathophysiology [1]. In general, a network disorder of the central nervous system involving cortical, thalamic, cerebellar and basal ganglia structures is considered to be the main cause of these pathological movement patterns [2].

Dystonia is now classified according to a two-axis system. The first axis describes the clinical presentation according to four different aspects: age at onset, topology, time-course of symptom development, and the presence of possible non-dystonic symptoms. The second axis of the classification scheme then refers to the etiology of the disease, distinguishing between inherited, acquired and idiopathic forms [1].

In addition to the motor phenotype, patients often complain of non-motor problems such as affective disorders, cognitive deficits, or pain [3]. Not least for this reason, dystonic diseases are often associated with a quality of life (QoL) that patients experience as significantly reduced [4].

Due to the heterogeneous clinical picture and the high disease burden that patients often suffer from, the treatment of dystonia remains a diagnostic and therapeutic challenge today [5]. Although used since the 1980s [6,7], chemical denervation via the injection of botulinum neurotoxin (BoNT) is still the therapy of choice for the treatment of focal or segmental dystonia such as orofacial (Meige syndrome (MS)) or cervical dystonia (CD) [8]. However, a significant proportion of patients on BoNT therapy experience primary or secondary treatment failure [9]. The reasons for this may be manifold and some remain elusive [10]. BoNT therapy is challenging; the correct selection of the muscles to be injected and the precise application of the neurotoxin are critical prerequisites for therapeutic success. However, some dystonic symptoms are not well-suited for selective chemodenervation treatment, such as tremulous dystonia or complex multiaxial dystonic movements. Another aspect is the problem of possible antibody formation during chronic therapy with BoNT due to its potential immunogenicity [11]. However, as a recent meta-analysis indicates that the estimated detection rate of neutralizing antibodies in dystonic diseases is only about 1%, the actual contribution of immunerrorogenicity to the overall problem of treatment refractoriness can only be assessed to a limited extent [12]. Furthermore, not everything is fully understood about antibody formation, but some of the factors thought to contribute to it are application-related, such as short injection cycles or high doses per injection, while other aspects are related to the toxin itself, such as the composition, production and storage of the substances [13].

As pharmacotherapy is often not a promising treatment alternative in these cases, there is a real need for effective treatment options for focal dystonia [14]. Deep brain stimulation (DBS) has become established in dystonia treatment guidelines as a fallback strategy in cases of complete treatment failure with BoNT [15]. While BoNT therapy is purely symptomatic and targets the effector organ muscle, DBS is acting on the brain and trying to normalize the network dysfunction considered to be responsible for dystonic movement disorders [16]. However, the superiority of this therapy has not (yet) been scientifically proven. DBS has been shown to improve symptom control in focal [17], segmental [18] and generalized [19] dystonia, but the specific question of whether DBS can be effective in patients who have failed BoNT therapy has only been addressed in a few studies. This review article is dedicated to a systematic literature search on this topic and to the question of to what extent the use of stereotactic brain surgery in these cases can really be considered evidence-based.

## 2. Results

### 2.1. Current Evidence from Meta-Analyses

For the 2018 Cochrane review by Rodrigues et al., 379 records in all were evaluated, of which 34 full-text articles ultimately remained for assessment [20]. However, only two studies that met the quality criteria of a randomized controlled trial were included in the meta-analysis (Kupsch et al., 2006, and Volkmann et al., 2014) [17,18]. Whereas the trial by Volkmann et al. exclusively included CD patients with explicit primary or secondary therapy failure under BoNT, in the study by Kupsch et al. only just under 40% of the participants were segmental dystonia patients with a predominant pattern of CD. A total of 35% of the patients recruited for this project also received BoNT therapy beforehand, although it is not entirely clear from the article whether these were predominantly or exclusively patients with segmental dystonia. However, the assumption is that the intersection here must be large. Common to both studies was a sham-controlled study design that evaluated the effectiveness of the symptom control of dystonia via DBS of the internal globus pallidus (GPi) in a double-blind manner, although the scales used to assess symptom severity at the primary 3 months endpoint differed: Volkmann et al. used the CD-specific Toronto Western Severity Spasmodic Torticollis Rating Scale (TWSTRS) [21], whereas Kupsch et al. resorted to the Burke–Fahn–Marsden Dystonia Rating Scale (BFMDRS) [22] measuring dystonic symptoms throughout the body. Due to the heterogeneous composition of the patient populations of the two studies, the authors of the meta-analysis did not perform a pooled evaluation of the effect sizes with regard to the treatment outcome. However, the respective primary outcome parameters can be found below in Table 1. In both studies, significantly better symptom control was achieved in the verum stimulation group than in the sham condition. A pooled analysis of adverse events did not provide meaningful results regarding the risk of adverse events (AEs) (relative risk (RR) 1.58, 95% 0.98 to 2.54; 2 RCTs, 102 participants) and tolerability (RR 1.86, 95% CI 0.16 to 21.57; 2 RCTs, 102 participants).

Fan et al. had a much broader scope in their meta-analysis published in 2021 [23]. Of almost 4000 articles screened, 103 studies were included in the analysis. However, a wide variety of study protocols (case-control, long-term follow-up, etc.) and a heterogeneous patient population (children, tardive dystonia, MS, etc.) were included here. The significance of these data must therefore be considered from certain points of view, which the authors also present accordingly. For example, studies on DBS of the nucleus subthalamicus (STN) can be found in the data alongside GPi-stimulated patients. It is worth noting that there were no group differences between GPi and STN stimulation in terms of symptom control and safety data. In addition, this analysis showed that the effectiveness of the procedures seems to be slightly better for focal dystonia than for segmental and generalized forms (*p* = 0.012). Another interesting point from this publication is that when the safety data were analyzed, there was a significantly higher rate of AEs in the secondary dystonia group compared to the hereditary and idiopathic forms of the disease (*p* < 0.005). However, these data only allow limited conclusions to be drawn about the significance of DBS as a therapeutic alternative for BoNT treatment failure, as the data set was not intended for this question and it is not known exactly how many BoNT non-responders were actually included in the focal dystonia group. 

Wang et al. focused on DBS in patients with MS in their 2019 meta-analysis [24]. They reported screening more than 3000 records for the paper and ultimately included 23 studies in the analysis. In contrast to the studies in cervical or segmental dystonia with a cervical focus and generalized dystonia, there are no randomized, sham-controlled and double-blind studies in MS and thus no level 1 or 2 evidence. Finally, 115 patients from 23 studies and 23 study centers were included in the analysis. In 17 patients, the electrodes were implanted in the STN; the rest received GPi stimulation. All studies were ultimately rated by the authors as level 4 evidence. Pooled analysis showed a statistically significant efficacy of DBS on symptom control in patients with MS in terms of a reduction in the BFMDRS movement score, for example, of 64.8% compared to the baseline 3–6 months after implantation (7.0 ± 4.9 to 3.6 ± 2.5, *p* = 0.000). Safety data were described here only anecdotally; severe adverse events (SAE) did not occur. One patient each with a postoperative hematoma and an electrode infection was reported.

A study already published in 2016 by the same author with less patient data yielded comparable results [25], so a detailed presentation will be omitted here. The existing meta-analyses in this area show that, firstly, there is a lack of evidence, especially in the field of MS, where there are no randomized and controlled trial data at all. The situation is slightly better for CD, but the total number of patients included in this type of study does not exceed 80. Secondly, at least at the current level of evidence, DBS appears to be an effective treatment option for patients with focal and segmental dystonia with a craniocervical distribution pattern. The next step will be to take a closer look at some of the selected trials, in particular to answer the question of the specific impact of prior BoNT therapy upon associated treatment failure.

### 2.2. Overview of Selected Trials of DBS for Focal or Segmental Craniocervical Dystonia in Patients with BoNT Failure

Table 1 shows an overview of relevant publications on DBS for focal dystonia in which explicit information on pre-treatment with BoNT was available. No follow-up data or long-term analyses are listed (i.e., evaluation of the primary endpoint within 12 months or less). Studies were only included if four or more patients were enrolled. The formal quality standards of the subsumed studies vary considerably. In addition to a few controlled, randomized and double-blind trials, both prospective protocols with partial single-blinding and retrospective case series are included.

**Table 1:** Overview of selected trials of DBS for focal or segmental craniocervical dystonia in patients with BoNT failure.

Study	Patients	BoNT	Protocol	DBS Site	Motor Endpoint	Results
Kupsch 2006 [18]	24 GD 16 SD	14 NR	SC RD DB	GPi	BFMDRS	−15.8 ± 14.1
					movement	vs. −1.4 ± 3.8
					3 mo.	*p* < 0.001
Kiss 2007 [26]	10 CD	10 SNR	PS SB	GPi	TWSTRS	14.7 ± 4.2
					severity	to 8.4 ± 4.4
					12 mo.	*p* = 0.003
Ostrem 2007 [27]	6 MS	4 NR	PS	GPi	BFMDRS	22 ± 8.3
					movement	to 6.1 ± 4.2
					6 mo.	*p* < 0.028
Pretto 2008 [28]	4 CD	4 NR	PS SB	GPi	TWSTRS	12.8
					severity	to 7.5
					6 mo.	(SD and p: n/a)
Jeong 2009 [29]	6 CD	6 NR	RS	GPi	TWSTRS	60.5 ± 3.6
					total	to 21.1 ± 13.2
					3 mo.	*p* = 0.016
Sensi 2009 [30]	11 SD (9 CD + MS)	11 NR	SB	GPi	BFMDRS	36.6 ± 12.7
					movement	to 23.3 ± 10.7
					6 mo.	*p* < 0.0001
Ghang 2010 [31]	11 MS	11 NR	RS	GPi	BRMDRS	24.5 ± 5.9
					movement	to 8.9 ± 7.7
					3 mo.	*p* < 0.001
Ostrem 2011 [32]	9 CD	8 SNR	PS SB	STN	TWSTRS	53.1 ± 2.6
					total	to 29.6 ± 5.5
					12 mo.	*p* < 0.001
Reese 2011 [33]	12 MS	9 NR	RS SB	GPi	BFMDRS	21.4 ± 3.2
					movement	to 12.4 ± 4.3
					3-6 mo.	*p* < 0.001
Skogseid 2011 [34]	8 CD	8 SNR	PS SB	GPi	TWSTRS	62 (60–70)
					total	to 27 (5–24)
					6 mo.	*p* < 0.05
Schjerling 2013 [35]	4 GD 7 CD 1MF	2 NR	CO RD DB	GPi + STN	BFMDRS	GPi: −9.1 ± 6.7
					movement	STN: −13.8 ± 4.2
					6 mo.	*p* = 0.08 (GPi vs. STN)
						*p* < 0.05 (GPi + STN)
Sobstyl 2014 [36]	6 MS	6 NR	PS	GPi	BFMDRS	23.7 ± 6.7
					movement	to 8.7 ± 2.5
					3 mo.	*p* = 0.028
Volkmann 2014 [17]	62 CD	55 SNR 6 PNR	SC RM DB	GPi	TWSTRS severity	−5.1 ± 5.1
						vs. −1.3 ± 2.4
					3 mo.	*p* = 0.0024
Horisawa 2018 [37]	16 MS	10 NR	SB	GPi	BFMDRS	15.5 (11.8–22.0)
					movement	to 2.5 (1.4–7.5)
					3 mo.	*p* < 0.001
Yin 2022 [38]	9 CD	4 NR	RS	STN	TWSTRS	47.9 ± 9.5
					total	to 7.3 ± 16.0
					12 mo.	*p* = 0.008

Abbreviations: GD: generalized dystonia, SD: segmental dystonia, CD: cervical dystonia, SF: secondary forms of dystonia, MF: multifocal dystonia, MS: Meige syndrome, NR: non-reponder, PNR: primary non-responder, SNR: secondary non-responder, SC: sham controlled, RD: randomized, DB: double-blind, SB: single-blind, PS: prospective study, RS: retrospective study, CO: cross-over, GPi: internal globus pallidus, STN: subthalamic nucleus, BFMDRS: Burke–Fahn–Marsden Dystonia Rating Scale, TWSTRS: Toronto Western Severity Spasmodic Torticollis Rating Scale, mo.: months, n/a: not applicable or not available.

A total of 15 trials explicitly reported on prior treatment with BoNT. Of the 140 patients with CD studied in these trials, at least 112 were reported to have received BoNT and were described as primary, secondary or undefined treatment failures. One limitation here is that treatment failure was not consistently defined in the studies cited. In most cases, only inadequate pretreatment with BoNT was generally reported, with no detailed information on symptom response in percentage, years of treatment, total number of sessions, or dosages. For example, a precise definition of this term might be suggested as follows: historical documentation of at least three subsequent injection cycles with less than 30% symptom improvement, prospective documentation of one injection cycle with less than 30% improvement in the TWSTRS at the study site by a botulinum toxin-experienced physician, serological proof of neutralizing antibodies against BoNT, or a negative extensor digitorum brevis injection test (in accordance with the study protocol of Volkmann et al. [17]). In MS, 49 of the 60 patients in the cited studies had been treated with BoNT prior to DBS surgery. This means that at least 80% of the patients presented here did not respond satisfactorily to BoNT therapy. In each of these trials, DBS showed treatment success in the form of a statistically significant reduction in motor scale scores compared with the baseline or, in the few controlled trials, a greater reduction in symptoms than the sham intervention. The effect sizes vary widely. A statistical analysis was not performed because of the heterogeneity of the data sources. For example, dystonic symptoms were only partially assessed in a blinded fashion. In addition, the data were collected at very different times after surgery, at 3, 6 or 12 months. Furthermore, different scales were used for cervical dystonia, which significantly limits the comparability of the data. Since most of the studies also included patients without prior BoNT therapy, it would not have been possible to draw valid conclusions about a pure collective of BoNT failures by pooling the study results.

Despite all the limitations due to the poor quality of the data, partly caused by the methods used, DBS of both the GPi and STN can lead to a reduction in dystonic symptoms for patients with focal and segmental craniocervical dystonia, even if prior chemodenervation yielded unsatisfactory results.

Some studies also examined secondary outcomes, with a regular focus on QoL, pain, and depressive moods. These data are summarized in Table 2. Depressive symptoms were assessed with Beck’s Depression Inventory (BDI) [39] in all cases, pain predominantly with the pain subscale of the TWSTRS, and quality of life with the SF-36 [40]. Only in one study was pain assessed with a visual analog scale (VAS) alone. The only exception for QoL was the use of the Global Rating Scale (GRS). For the SF-36, it should be noted that not every study reported results on all eight domains asked about in the questionnaire. In some cases, only the physical functioning and mental health subcategories were reported.

Whenever assessed, the BDI score remained stable during the first months of stimulation. However, the occasional occurrence of depressive symptoms in terms of AEs will be reported in the next subsection. In most studies involving CD patients, a statistically significant reduction in pain was achieved, as evidenced by a reduction in the TWSTRS pain subscore. Since pain is virtually absent as a symptom in MS, it has not been systematically recorded in the corresponding trials. As patients’ QoL is often significantly impaired by dystonia, the measurement of this construct is of particular importance in therapeutic studies in this field. In most of the studies cited here, there was a significant improvement in the patients’ assessment of QoL during the observation period, which could be objectified using appropriate questionnaires. Interestingly, in the largest study on CD by Volkmann et al., the QoL questions of the SF-36 did not improve from the baseline within the 3-month blinded-treatment phase. At six months, however, the authors were able to link the CDQ-24 [41], another dystonia-specific QoL questionnaire, to a statistically significant improvement (*p* < 0.0001) [17]. 

In summary, most studies of CD achieved the secondary endpoints of pain reduction and improvement in QoL when they were evaluated. For MS, no valid statement can be made in this regard. 

### 2.3. Safety and Tolerability Aspects

To provide a good overview of the safety data, the frequency of AEs and SAEs, where reported in the publications, has been entered in Table 3. The most commonly reported device-related AEs, lead infection and lead dislocation, were listed numerically. This also applies to the most common stimulation-related side effects such as dysarthria, dyskinesia, depression and worsening of pre-existing dystonic symptoms. Other surgical complications occasionally reported include lead edema, postoperative subdural compartment, seizures, or problems due to inadequate electrode cable length. Other effects triggered by the stimulation included dysphagia, balance difficulties, gait disturbance and fine motor problems. Stimulation-related effects were mostly described as mild by the authors of the studies and were often modifiable by changing the stimulator settings. A specific review of the 23 SAEs reported here shows that at least 17 of these were considered to have been resolved within the studies, so, at least in these cases, no permanent patient harm is likely. 

In summary, the rate of persistent surgical sequelae appears to be less than one percent, which is also consistent with the assumption that the perioperative risk in dystonia patients is lower than in Parkinson’s disease (PD) due to the younger average age of dystonic patients at the time of surgery. In PD, the incidence of permanent morbidity from surgery is about 1% [42]. As a limiting factor, the AE reporting in retrospective studies may be less accurate than in prospective studies. When evaluating these data, this should be taken into account.

## 3. Discussion

The purpose of this review was to address the specific question of whether, in light of published data on the use of DBS in patients with focal and/or segmental dystonia with a craniocervical focus, DBS is an effective therapeutic modality specifically in patients who have failed prior BoNT therapy. This question is relevant, because levodopa-responsiveness—i.e., the efficacy of pharmacotherapy—is the most important predictor of outcome in DBS for advanced Parkinson’s disease. Of course, due to the completely different pathophysiology, a direct comparison between the diseases is not possible. However, it should be noted that a fundamental problem of toxin therapy, namely immunogenicity, was one of the main driving forces behind the use of the innovative treatment method of DBS, even in focal dystonia.

As mentioned earlier, there is little literature that provides explicit information on how dystonia patients were treated prior to DBS surgery. In addition, there are few randomized, controlled, double-blind trials in this area, so data on the efficacy of DBS for focal and segmental dystonia must be derived in part from retrospective studies. However, for CD, at least one study with the highest level of evidence demonstrates efficacy in patients with primary and secondary treatment failure after BoNT treatment, with a precise definition of the term therapy response as described above [17]. With 62 patients enrolled, this is a respectable number for a rare disease and a complex treatment. All of the studies listed here showed a statistically positive effect in controlling dystonic symptoms. Methodological limitations such as unblinded assessment and retrospective data collection reduce the validity of the data. In addition, publication bias is likely. Nevertheless, the effect sizes are sometimes very convincing; moreover, in the majority of studies, the patients’ subjectively perceived QoL also improved with the use of DBS. The same is true for pain reduction in patients with CD. 

In terms of safety, the rate of permanent surgical sequelae was within the range expected from preliminary studies in Parkinson’s disease. Stimulation-related side effects are generally described by the study authors as mild and easily controlled. Thus, in the context of the data, the safety profile appears to be expected with respect to known procedure-related issues and thus appropriate for the intervention.

Overall, the level of evidence for DBS therapy in dystonia is not high, but the available studies, combined with clinical experience, support the assumption that the treatment is effective. Based on the high proportion of patients in the study who received BoNT as a pretreatment, the question of efficacy in treatment failure also appears to be answered in the affirmative. Only further randomized, controlled and double-blind trials could provide more evidence. However, these will probably never be performed due to the effort and cost involved, especially since DBS therapy for treatment failure has already been included in the relevant guidelines as a second-line option.

However, one very important point has been neglected in this work, as well as in clinical routines and scientific evaluation. Currently, in light of the data presented here and in accordance with the guidelines, DBS is used primarily in patients who report no or little benefit from BoNT therapy. However, we do know that a significant number of patients are partial responders to BoNT therapy but still fail to achieve satisfactory symptom control. Only about half of patients on long-term BoNT therapy have sufficient symptom relief for twelve weeks or longer [43]. In addition to latency to onset, wearing-off in symptom control is an everyday clinical problem well known to users and patients. Since this circumstance is certainly due in part to the intrinsic pharmacological properties of BoNT and inevitable variations in the mode of application, even the best clinical practice may not provide a complete solution to this problem. In plain language, this means that, even though patients are considered to be BoNT responders, they may regularly suffer from inadequate symptom control for a few weeks each quarter. The negative consequences for patients in terms of non-motor symptoms such as affective disorders, and the associated reduction in perceived QoL due to poor symptom control, have been previously exposed. However, there are also socioeconomic consequences for patients, such as a reduction in the ability to work, with a significantly higher incidence of early retirement compared to the general population [44]. Based on the physical principle of action, it is reasonable to assume that significantly more sustained symptom control can be achieved with DBS treatment than with injection treatment. This situation raises the question of whether DBS should be offered earlier as an equivalent alternative therapy for patients with a partial response to BoNT, at least if the patient requests it. It is possible that optimizing symptom control earlier in the disease course could also be associated with a better impact on QoL and socioeconomic outcomes. This question is currently being addressed by a prospective, randomized, double-blind, double-dummy-design study to evaluate the best clinical application of GPi-DBS and BoNT therapy in a head-to-head comparison in patients with CD with a partial BoNT response [45]. The so-called StimTox-CD study is currently recruiting in Germany and has already randomized nearly 40 patients. The first study results are expected during 2025. If the superiority of DBS in terms of symptom control and improvement of QoL parameters is proven, a paradigm shift in the treatment of patients with CD could be considered if the safety data are acceptable. Depending on patient preference, DBS could then be offered as an equal first-line alternative for CD patients who respond to BoNT.

## 4. Materials and Methods

Based on recent meta-analyses on the topic of DBS in dystonic disorders [20,23,24,25], the specific question for this review was: is DBS an effective treatment modality for patients with primary or secondary BoNT failure in dystonia with a predominant craniocervical distribution pattern? The focus was therefore on focal or segmental forms of dystonia, such as CD and MS, because only in these variants is BoNT regularly used as a mono- or primary therapy. For these reasons, generalized dystonia, hemidystonia and tardive dystonia were not primarily included in the review. In addition, only trials that included patients who had previously received BoNT, and in which the study description gave details of the therapeutic success of BoNT, were selected, so that a statement could be made in terms of the research question. We also did not include head-to-head comparisons between STN and GPi unless a cumulative symptom-reduction efficacy compared to the baseline was reported.

## Figures and Tables

**Table 2:** Secondary Outcome Parameters.

Study	Depression	Pain	Quality of Life
Kupsch, 2006 [18]	**BDI**	**VAS**	**SF-36**
	***p* = 0.008**	***p* < 0.001**	***p* < 0.001 (P), *p* = 0.01 (M)**
Kiss, 2007 [26]	**BDI**	**TWSTRS pain**	**SF-36**
	***p* < 0.001**	***p* < 0.001**	***p* = 0.003**
Ostrem, 2007 [27]	n/a	TWSTRS pain	n/a
		*p* = 0.08	
Pretto, 2008 [28]	n/a	n/a	GRS
			n/a
Jeong, 2009 [29]	n/a	TWSTRS pain	n/a
		*p* = 0.006	
Sensi, 2009 [30]	n/a	n/a	n/a
Ghang, 2010 [31]	n/a	n/a	n/a
Ostrem, 2011 [32]	BDI	TWSTRS pain	SF-36
	n.s.	*p* = 0.005	***p* = 0.011 (P), *p* = 0.028 (M)**
Reese, 2011 [33]	n/a	n/a	n/a
Skogseid, 2011 [34]	BDI	TWSTRS pain	**SF-36**
	NS	*p* < 0.05	***p* = 0.018–0.028 (M, P, etc.)**
Schjerling, 2013 [35]	n/a	n/a	SF-36
			*p* = 0.01 (P), n.s. (M)
Sobstyl, 2014 [36]	n/a	n/a	n/a
Volkmann, 2014 [17]	BDI	TWSTRS pain	SF-36
	*p* = 0.02	*p* = 0.47	*p* = 0.27
Horisawa, 2018 [37]	n/a	n/a	n/a
Yin, 2022 [38]	n/a	TWSTRS pain	SF-36
		*p* = 0.008	***p* = 0.001–0.008 (M, P, etc.)**

Abbreviations: BDI: Beck Depression Inventory, VAS: Visual Analog Scale, SF-36: Short Form 36 (subdomain P: physical functioning, subdomain M: mental health), TWSTRS: Toronto Western Severity Spasmodic Torticollis Rating Scale, GRS: Global Rating Scale, n/a: not applicable or not available.

**Table 3:** Safety and tolerability aspects.

Study	AE Total	SAE Total	Lead Infection	Lead Dislocation	Dysarthria	Dyskinesia	Dystonia Worsening	Depression
Kupsch 2006 [18]	22	5	4	1	5	5	0	0
Kiss 2007 [26]	4	0	0	0	0	0	0	0
Ostrem 2007 [27]	4	0	0	0	0	0	0	0
Pretto 2008 [28]	2	0	0	0	0	0	0	0
Jeong 2009 [29]	n/a	n/a	0	0	3	0	1	0
Sensi 2009 [30]	6	1	0	1	2	2	0	0
Ghang 2010 [31]	n/a	0	0	0	+	0	0	+
Ostrem 2011 [32]	39	0	0	0	2	9	0	5
Reese 2011 [33]	n/a	0	0	0	+	0	0	0
Skogseid 2011 [34]	n/a	1 *	0	0	+	+	0	0
Schjerling 2013 [35]	n/a	n/a	2	0	0	2	0	3
Sobstyl 2014 [36]	1	0	0	0	0	0	0	0
Volkmann 2014 [17]	41	16	2	2	7	3	3	4
Horisawa 2018 [37]	n/a	n/a	0	0	3	5	2	3
Yin 2022 [38]	17	0	0	0	0	8	0	0

* Electrode oedema. Abbreviations: AE: adverse events, SAE: severe adverse events, n/a: not applicable or not available.

## Data Availability

Not applicable.
